# Diazomethane umpolung atop anthracene: an electrophilic methylene transfer reagent†
†Electronic supplementary information (ESI) available: Experimental procedures and characterization data. CCDC 1580349–1580351. For ESI and crystallographic data in CIF or other electronic format see DOI: 10.1039/c7sc04506a


**DOI:** 10.1039/c7sc04506a

**Published:** 2017-12-21

**Authors:** Maximilian Joost, Wesley J. Transue, Christopher C. Cummins

**Affiliations:** a Department of Chemistry , Massachusetts Institute of Technology , Cambridge , MA 02139 , USA . Email: ccummins@mit.edu

## Abstract

Formal addition of diazomethane's terminal nitrogen atom to the 9,10-positions of anthracene yields H_2_CN_2_**A** (**1**, **A** = C_14_H_10_ or anthracene). The synthesis of this hydrazone is reported from Carpino's hydrazine H_2_N_2_**A** through treatment with paraformaldehyde. Compound **1** has been found to be an easy-to-handle solid that does not exhibit dangerous heat or shock sensitivity. Effective umpolung of the diazomethane unit imbues **1** with electrophilicity at the methylene carbon center. Its reactivity with nucleophiles such as H_2_CPPh_3_ and N-heterocyclic carbenes is exploited for C=C bond formation with elimination of dinitrogen and anthracene. Similarly, **1** is demonstrated to deliver methylene to a nucleophilic singlet d^2^ transition metal center, W(ODipp)_4_ (**2**), to generate the robust methylidene complex [**2**=CH_2_]. This behavior is contrasted with that of the Wittig reagent H_2_CPPh_3_, a more traditional and Brønsted basic methylene source that upon exposure to **2** contrastingly forms the methylidyne salt [MePPh_3_][**2**≡CH].

## Abstract 2

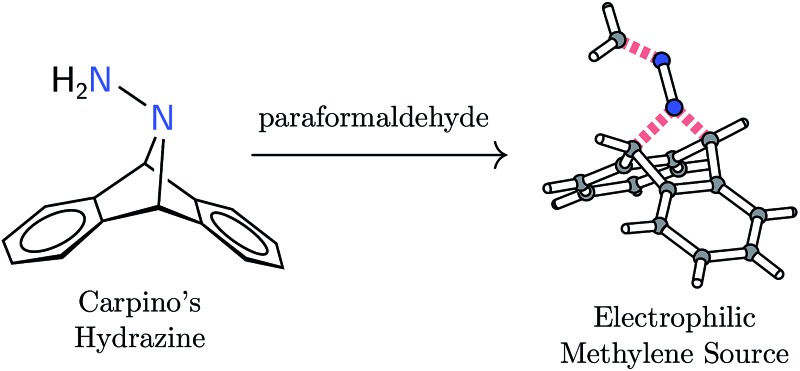
Formal addition of diazomethane's terminal nitrogen atom to the 9,10-positions of anthracene yields H_2_CN_2_**A** (**1**, **A** = C_14_H_10_ or anthracene).

Diazomethane is infamous for the dangers associated with its use.^1^ Despite its synthetic versatility, diazomethane's high toxicity and propensity to explode should give a chemist pause before committing to its use. In an effort to offer an alternate methylene source using an anthracene-based strategy,^2^–^8^ we report herein the synthesis and some initial reactivity studies of H_2_CN_2_**A** (**1**, **A** = C_14_H_10_ or anthracene), a molecule conceived as a formal adduct between diazomethane and anthracene. An initial survey of the reactivity patterns of **1** has revealed it not to be a simple substitute for diazomethane, instead characterizing it as a unique electrophilic methylene source. Its electrophilicity differentiates **1** from common metal-free methylene transfer reagents such as diazomethane and methylene triphenylphosphorane.

Synthesis of hydrazone **1** proceeded from Carpino's hydrazine H_2_N_2_**A** upon paraformaldehyde treatment in a biphasic diethyl ether–water mixture,^9^,^10^ providing the target molecule in 74% isolated yield (Scheme 1). An X-ray diffraction study of its structure revealed expected metrical data.^11^

**Scheme 1:**
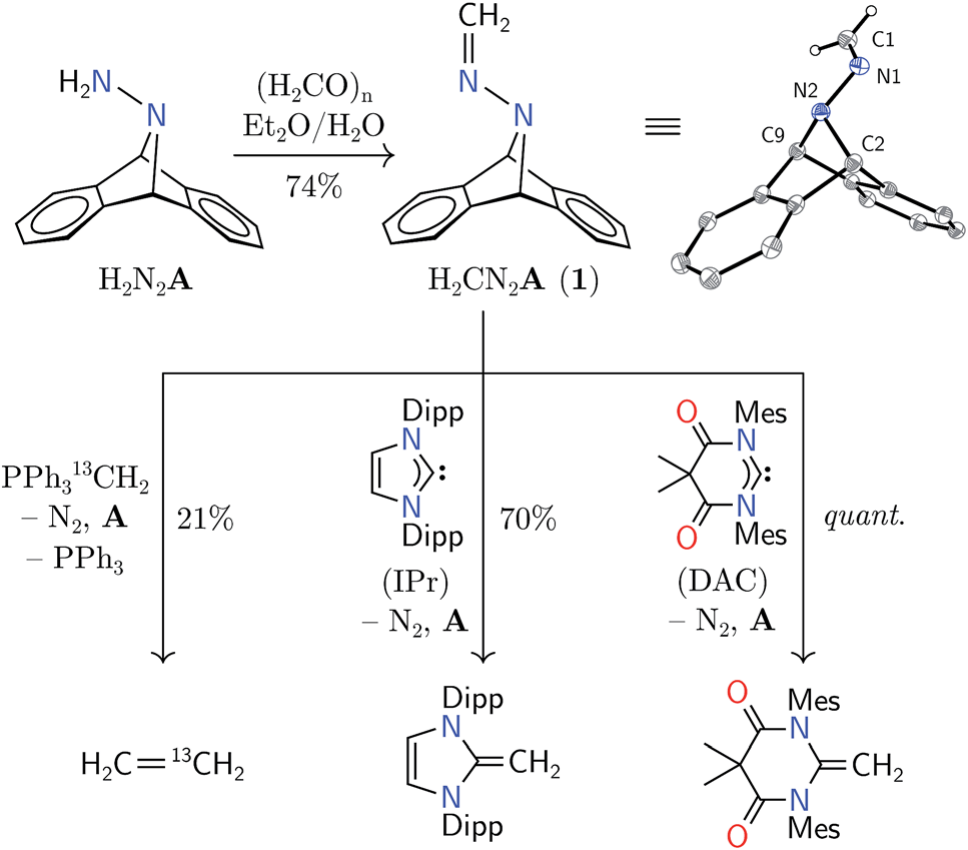
Synthesis of methylene hydrazone **1** and initial studies of methylene transfer (Mes = mesityl, Dipp = 2,6-diisopropylphenyl), shown alongside its structure from an X-ray diffraction study. Thermal ellipsoids are shown at the 50% probability level. Selected distances [Å] and angles [°]: N1–C1 1.275(2), N1–N2 1.389(1), N2–C2 1.508(1), N2–C9 1.521(2); N2–N1–C1 118.3(1).

Hydrazone **1** was found to be an air-stable and crystalline solid, easily manipulable and displaying no propensity for detonation upon heating or shock. The solid was found to be volatile by thermogravimetric analysis (TGA), which showed gradual sample evaporation up to 120 °C without any discrete mass-loss events that would be expected from its fragmentation into diazomethane and anthracene. Within a sealed capillary, **1** melted without explosion (116–119 °C). After heating the melt to 140 °C, NMR spectroscopic analysis of the resolidified solid showed 74% recovery of **1** with 26% anthracene production. Its behavior in solution was similar, evincing only slow fragmentation into anthracene at temperatures greater than 120 °C. The volatility of this compound foiled attempts at analysis of its thermal behavior by molecular beam mass spectrometry (MBMS), limiting our ability to comment on the fragments directly produced by its thermal fragmentation.^2^–^5^

Having established **1** to pose a low explosion risk, we were encouraged to proceed to test its reactivity as a methylene synthon. Our initial investigations rapidly uncovered contrasting reactivity patterns *vis-à-vis* those characteristic of diazomethane. For example, methylation of carboxylic acids, a hallmark of diazomethane reactivity,^12^ did not proceed upon treatment with excess pivalic acid, acetic acid, or trifluoroacetic acid. These experiments were informative, and led us to consider more closely the electronic structure of **1**.

Hydrazones are known to be carbon ambiphiles;^13^ however, **1** did not demonstrate nucleophilicity. Such behavior is not unexpected, as the π_CN_ is known to be polarized away from the carbon center, although less so than an imine π_CN_ or a ketone π_CO_ bond.^14^ The polarization of this bond suggests that **1** should be expected to exhibit moderate electrophilicity at its methylene carbon. This would effectively induce umpolung of the diazomethane unit as diazomethane generally reacts as a carbon nucleophile.^15^

The predicted reversal of philicity was initially confirmed by successful methylene transfer in the reaction between **1** and H_2_CPPh_3_. Combination of these two reagents in benzene-d_6_ yielded ethylene in 21% yield over 12 h in concert with anthracene, triphenylphosphine, and, presumably, dinitrogen. The reaction was found to produce several unidentified byproducts by NMR spectroscopy, explaining the low yield of ethylene; however, isotopic labelling of the ylide led to H_2_C= ^13^CH_2_ from **1** and H_2_^13^CPPh_3_, and H_2_C=CD_2_ from **1** and D_2_CPPh_3_, confirming ethylene formation through the unification of the electro- and nucleophilic methylene units. Although the yield was low, this mode of reactivity was instructive for our further studies.

The electrophilicity of **1** lent itself well to the synthesis of N-heterocyclic olefins from N-heterocyclic carbenes (NHCs).^16^ In benzene-d_6_ solution, **1** reacted with nucleophilic IPr (1,3-bis(2,6-diisopropylphenyl)imidazol-2-ylidene) to yield the corresponding olefin in 70% yield after 13 h at 80 °C.^16^ As a nucleophile with increased electrophilicity, the Bielawski *N*,*N*′-diamidocarbene (“DAC”) was found to react in essentially quantitative yield to form a new C=C bond over 24 h at 22 °C.^17^ This mode of reactivity differs markedly from that of diazoalkanes, which have been documented to react with NHCs at their electrophilic N-terminus to produce azines with a new C=N–N=C moiety.^18^ Heating **1** with triphenylphosphine or tricyclohexylphosphine has not yielded the analogous yieldes, suggesting a modest Lewis acidity at the carbon center of **1**.

It is rare for diazomethane to be used in transition metal chemistry for the synthesis of a stable methylidene complex.^19^ In fact, the use of diazoalkanes in d-block chemistry is often complicated by their propensity for side reactions other than alkylidene delivery.^20^,^21^ The reactivity differences between **1** and diazomethane thus encouraged us to attempt the use of **1** in methylidene complex synthesis to see if engagement of the terminal nitrogen in bonding to anthracene subdues deleterious alternate reaction pathways.

We identified [W(ODipp)_4_] (**2**, ODipp = 2,6-diisopropylphenoxide)^22^,^23^ as a d^2^ transition metal complex well poised to behave as a methylene acceptor.^24^ Complex **2** is synthetically easy to access, and its square-planar geometry features a nucleophilic lone pair of electrons housed in a metal-centered d_*z*^2^_-like orbital, analogously to related tantalum and molybdenum singlet d^2^ species.^8^,^25^ Treatment of **2** with excess **1** gave facile formation of the anticipated methylidene complex [**2**=CH_2_] after mild heating in benzene to 55 °C for 35 h (Scheme 2). Characteristically deshielded proton and carbon resonances of the CH_2_ unit were found by NMR spectroscopy: ^1^H *δ* 8.95 ppm and ^13^C *δ* 232.9 ppm with scalar coupling constants of ^2^*J*_WH_ = 156.0 Hz, ^1^*J*_WC_ = 185.0 Hz, and ^1^*J*_CH_ = 155.6 Hz. The ^1^*J*_CH_ coupling constant was typical of metal alkylidenes lacking significant agostic character.^26^–^29^ The success of **1** in this capacity was exciting, as the rarity of terminal, isolable methylidene complexes^30^ makes new methods for their generation welcome developments.

**Scheme 2:**
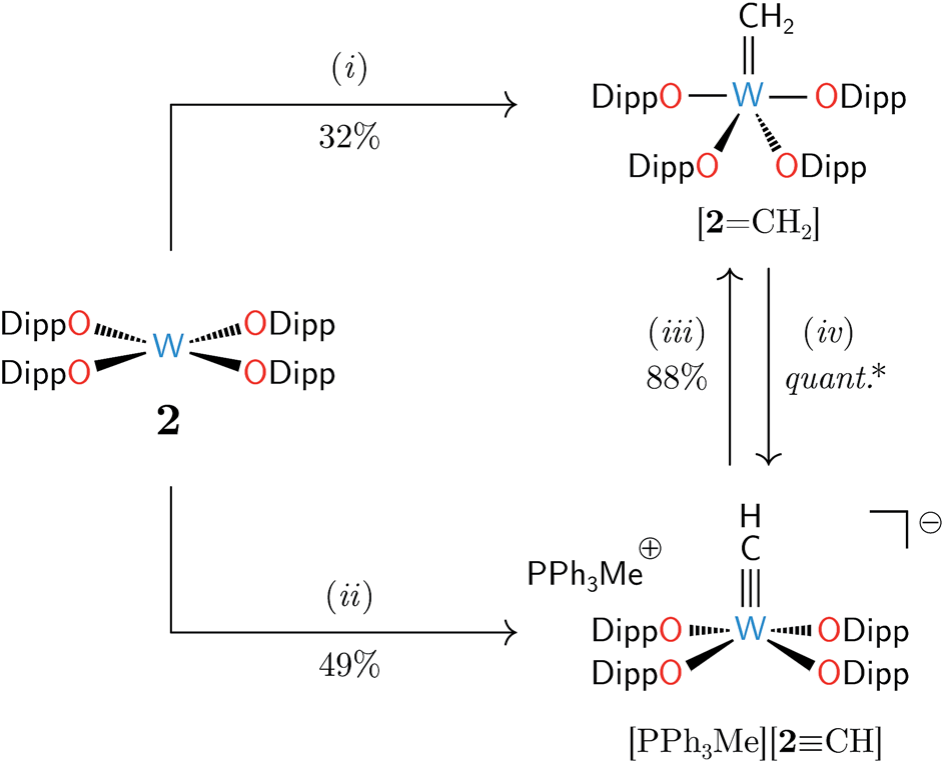
Comparative reactivity of W(ODipp)_4_ (**2**): (i) H_2_CN_2_**A** (**1**, 10 equiv.), benzene, 55 °C, 35 h; (ii) H_2_CPPh_3_ (2.0 equiv.), THF, 25 °C, 30 min; (iii) lutidinium triflate (1.0 equiv.), THF, 25 °C, 5 min; (iv) PPh_3_CH_2_ (1.0 equiv.), THF, 25 °C, 30 min. (*) NMR spectroscopic analysis showed (iv) to be quantitative.

Crystallization from pentane at –35 °C overnight enabled an X-ray diffraction study of [**2**=CH_2_] (Fig. 1, left) that confirmed the molecular structure. Although the data were not of high quality, the coordination geometry about the tungsten center was unambiguously identified to be intermediate between square pyramidal and trigonal bipyramidal (*τ* = 0.48),^31^ and the alkylidene bond was identified with a W···C interatomic distance of 1.864(4) Å. This bond length is typical of a W=C double bond^32^ and similar to values reported for other tungsten(vi) methylidenes.^29^,^33^–^35^ Compound [**2**=CH_2_] was not found to react productively with ethylene or 1-hexene upon heating to 70 °C in benzene-d_6_ for 18 h, confirmed by a lack of isotopic migration from [**2**= ^13^CH_2_] to the olefins.^36^ Under these conditions, [**2**=CH_2_] also did not react with mesitaldehyde or 4,4′-dimethylbenzophenone to form [**2**≡O] and the corresponding olefins. Despite this, [**2**=CH_2_] is notable as an example of a methylidene complex with aryloxides as the exclusive supporting ligands. As such, it is an interesting structural model for methylidene complexes supported by silica or alumina surfaces implicated in alkane or olefin metathesis.^37^–^39^

**Fig. 1:**
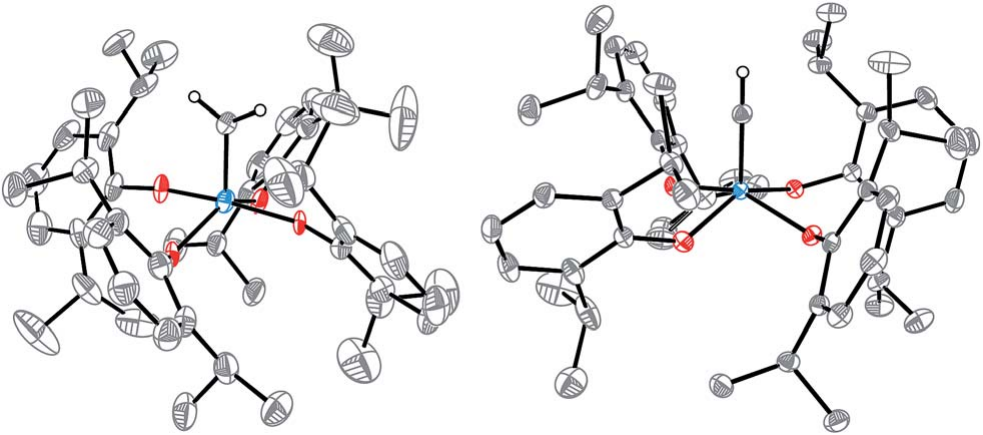
Molecular structures of (left) [**2**=CH_2_] and (right) [MePPh_3_][**2**≡CH] from single-crystal X-ray diffraction studies shown with thermal ellipsoids at the 50% probability level. All hydrogen atoms except for the methylidene and methylidyne hydrogens are omitted for clarity, as is the [MePPh_3_] cation. Interatomic distances for tungsten-carbon multiple bonds: (left) W1=C1 1.864(4) Å, (right) W1≡C1 1.749(1) Å.

The reactivity of **1** was particularly satisfying after discovery of the contrasting behavior of H_2_CPPh_3_, a known reagent for CH_2_ delivery to transition metal centers.^40^–^42^ Treating a solution of **2** with H_2_CPPh_3_ (1 equiv.) in THF at 25 °C rapidly consumed 50% of **2** and formed the methylidyne salt [MePPh_3_][**2**≡CH]. Doubling the amount of H_2_CPPh_3_ gave total consumption of **2** and provided [MePPh_3_][**2**≡CH] in 49% isolated yield (Scheme 2). Variation of the stoichiometry and temperature of this reaction did not lead to conditions for [**2**=CH_2_] formation, indicating competitive deprotonation of intermediate [**2**=CH_2_] by Brønsted basic H_2_CPPh_3_. Such acid-base chemistry is postulated to play a critical role in the formation of surface-bound alkylidenes and alkylidynes for alkane and olefin metathesis,^37^,^38^ meaning [**2**=CH_2_] serves also as an interesting reactivity model for alkylidyne synthesis mediated through proton transfer. This was corroborated by independent deprotonation of [**2**=CH_2_] with H_2_CPPh_3_, and highlights the utility of **1** as a weakly Brønsted basic source of methylene. Protonation of [MePPh_3_][**2**≡CH] using lutidinium triflate presents a complementary route to [**2**=CH_2_].

An X-ray crystallographic study of [MePPh_3_][**2**≡CH] revealed a W···C interatomic distance of 1.749(1) Å and a square pyramidal (*τ* = 0.21) coordination geometry about tungsten. A search of the CSD revealed this to be the first catalogued example of a structurally characterized metal methylidyne in an all-oxygen ligand environment, and the first catalogued example of a tungsten(vi) methylidyne complex.

As interest in metal methylidene species is rapidly growing both in homogeneous and heterogeneous catalysis,^37^–^39^,^43^–^45^ we hope **1** can be further exploited in their syntheses. Compound **1** has also shown promise in formation of new C=C bonds with H_2_CPPh_3_ and NHCs, and may find use in construction of terminal olefins.

## Conflicts of interest

There are no conflicts to declare.

## Supplementary Material

Supplementary information

Crystal structure data
