# Supplementary material for: Diazomethane umpolung atop anthracene: an electrophilic methylene transfer reagent
Source: Chem Sci. 2017 Dec 21;9(6):1540–3. doi: 10.1039/c7sc04506a (PMC5890322; doi:10.1039/c7sc04506a)
Supplement: Supplementary file 1 [file SC-009-C7SC04506A-s001.pdf]

Electronic Supplementary Material (ESI) for Chemical Science.
This journal is © The Royal Society of Chemistry 2017

Diazomethane Umpolung atop Anthracene: An Electrophilic Methylene Transfer Reagent

Maximilian Joost, Wesley J. Transue, Christopher C. Cummins*

Supporting Information

Table of Contents

S.1 Synthetic Details and Characterization of Products **S.2**

S.1.1 General Information . . . S.2

S.1.2 Synthesis of H_2CN_2**A** (**1**) . . . S.3

S.1.3 Reaction of **1** with H_2CPPh_3 . . . S.6

S.1.4 Reaction of **1** with *N*-Heterocyclic Carbenes . . . S.6

S.1.5 Synthesis of **2**=CH_2 . . . S.9

S.1.6 Synthesis of [$MePPh_3$][**2**≡CH] . . . S.11

S.2 X-Ray Diffraction Studies **S.14**

References **S.17**

S.1 Synthetic Details and Characterization of Products

S.1.1 General Information

Unless otherwise indicated, all manipulations were performed in a Vacuum Atmospheres model MO-40M glovebox under an inert atmosphere of purified N_2. All solvents were obtained anhydrous and oxygen-free by bubble degassing (Ar), purification through columns of alumina and/or Q5,[1] and storage over molecular sieves.[2] Deuterated solvents were purchased from Cambridge Isotope Labs, then degassed and stored over molecular sieves for at least 2 days prior to use. Celite 435 (EM Science) and activated charcoal were dried by heating above 250 °C under dynamic vacuum for at least 48 h prior to use. Glassware was oven-dried for at least three hours at temperatures greater than 150 °C. 7-Amino-2,3:5,6-dibenzo-7-azabicyclo[2.2.1]hepta-2,5-diene (H_2N_2**A**, **A** = 9,10-dihydroanthracene-9,10-diyl) was prepared as previously described by Carpino and coworkers.[3] 1,3-Bis(2,6-diisopropylphenyl)imidazol-2-ylidene (IPr) was purchased from Aldrich. *N*,*N'*-dimesityl-4,6-diketo-5,5-dimethylpyrimidin-2-ylidene (DAC) was prepared as described by Bielawski and coworkers.[4] Tungsten tetrakis(2,6-diisopropylphenolate), [W(ODipp)$_4$] (**2**), was prepared as previously described by Schrock and coworkers.[5] Methylene triphenylphosphorane, $H_2C{=}PPh_3$, was prepared using the procedure of Bestmann.[6] Where mentioned, room temperature indicates 22–24 °C.

NMR spectra were obtained on Varian Inova 300 and 500 instruments equipped with Oxford Instruments superconducting magnets, on a Jeol ECZ-500 instrument equipped with an Oxford Instruments superconducting magnet, or on a Bruker Avance 400 instrument equipped with a Magnex Scientific or with a SpectroSpin superconducting magnet. ^{1}H NMR and ^{13}C NMR spectra were referenced internally to residual solvent signals,[7] and ^{31}P NMR spectra were referenced externally to 85% aqueous H_3PO_4.

Attenuated total reflection infrared (ATR-IR) spectra were recorded on a Bruker Tensor 37 Fourier transform IR (FTIR) spectrometer. Elemental analyses were performed by Robertson Microlit Laboratories (Ledgewood, NJ, USA).

S.1.2 Synthesis of H_2CN_2A (1)

The preparation of 7-methaneimino-2,3:5,6-dibenzo-7-azabicyclo[2.2.1]hepta-2,5-diene (**1**, H_2CN_2**A**) was performed under air employing ACS reagent grade solvents. Under ambient conditions, the starting material Carpino's hydrazine H_2N_2**A** is a stable reagent and can be stored under air for extended periods of time.

A biphasic mixture of H_2N_2**A** (500 mg, 2.40 mmol, 1.00 equiv) and paraformaldehyde (86.5 mg, 2.88 mmol, 1.20 equiv) in diethyl ether (20 mL) and water (20 mL) was stirred vigorously for 12 h at room temperature. Phases were separated, and the aqueous phase was extracted with diethyl ether (2 × 20 mL). The combined organic layers were dried over $MgSO_4$ and filtered through a Celite/charcoal pad (diameter *ca.* 2 cm, height *ca.* 3 cm). The plug was rinsed with additional ether (70 mL). Volatiles were removed from the colorless filtrate to give **1** as a colorless solid (Yield: 390 mg, 1.77 mmol, 74%).

Elem. Anal. Found(Calc'd) for $C_{15}H_{12}N_2$: C 81.56 (81.79), H 5.32 (5.49), N 12.71 (12.72); Melting point: 116-119 °C (dec.; color change from colorless to orange.); ^{1}H NMR (benzene-d_6, 400 MHz, 20 °C) δ 7.05 (m, 4H, H_{ar}), 6.81 (m, 4H, H_{ar}), 6.30 (d, $^2J_{HH}$ = 11.6 Hz, 1H, $H_{methylene}$), 6.19 (d, $^2J_{HH}$ = 11.6 Hz, 1H, $H_{methylene}$), 5.44 (s, 2H, $H_{bridgehead}$); ^{1}H NMR (chloroform-d, 500 MHz, 20 °C) δ 7.35 (m, 4H, H_{ar}), 7.01 (m, 4H, H_{ar}), 6.79 (d, $^2J_{HH}$ = 11.6 Hz, 1H, $H_{methylene}$), 6.53 (d, $^2J_{HH}$ = 11.6 Hz, 1H, $H_{methylene}$), 5.70 (s, 2H, $H_{bridgehead}$); $^{13}C\{^1H\}$ NMR (chloroform-d, 126 MHz, 20 °C) δ 145.8 (s, C_{ipso}), 136.1 (s, $C_{methylene}$), 126.2 (s, CH_{ar}), 122.2 (s, CH_{ar}), 71.1 (s, $CH_{bridgehead}$); IR (ATR, cm^{-1}): 3050, 3029, 2923, 2854, 1677, 1593, 1480.

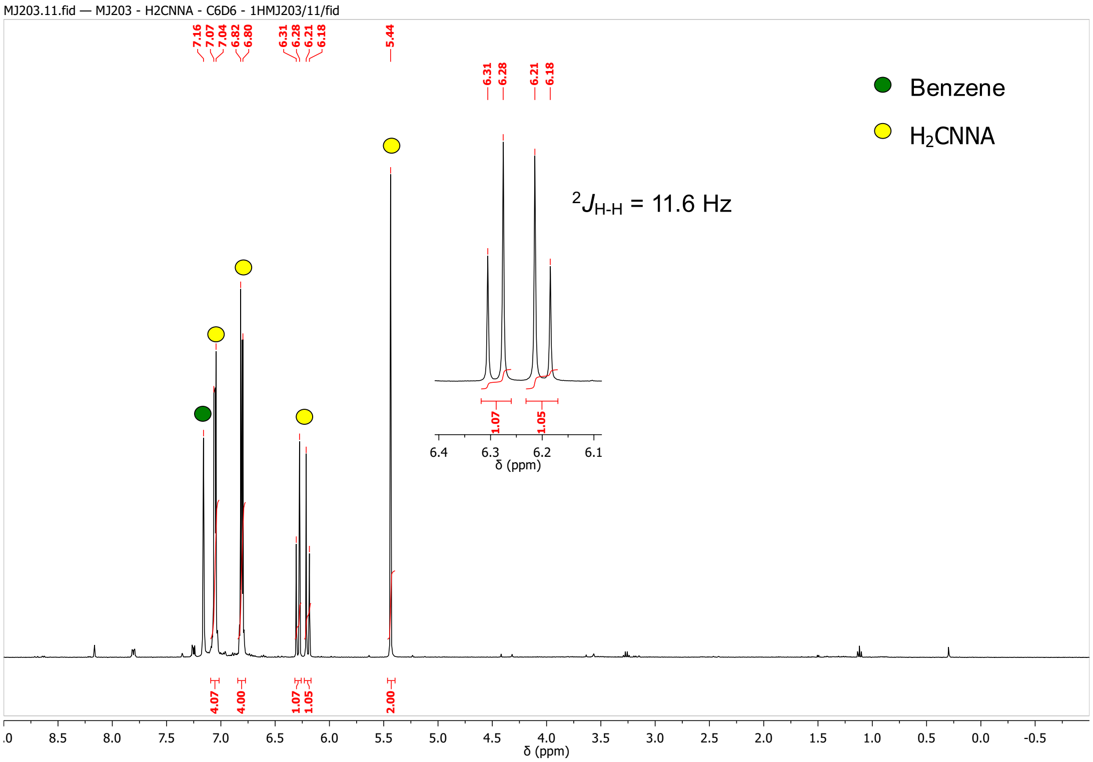

Figure S.1: ^{1}H NMR spectrum (benzene-d_6, 400 MHz, 20 °C) of **1**.

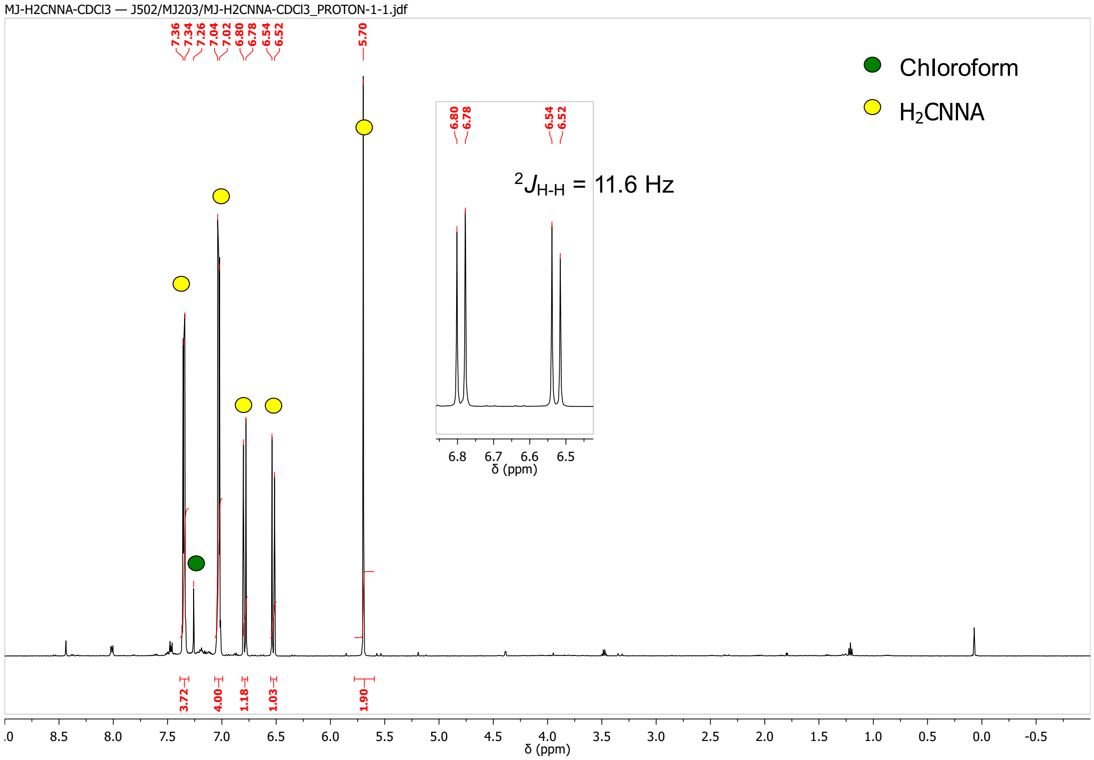

Figure S.2: ^{1}H NMR spectrum (chloroform-d, 500 MHz, 20 °C) of **1**.

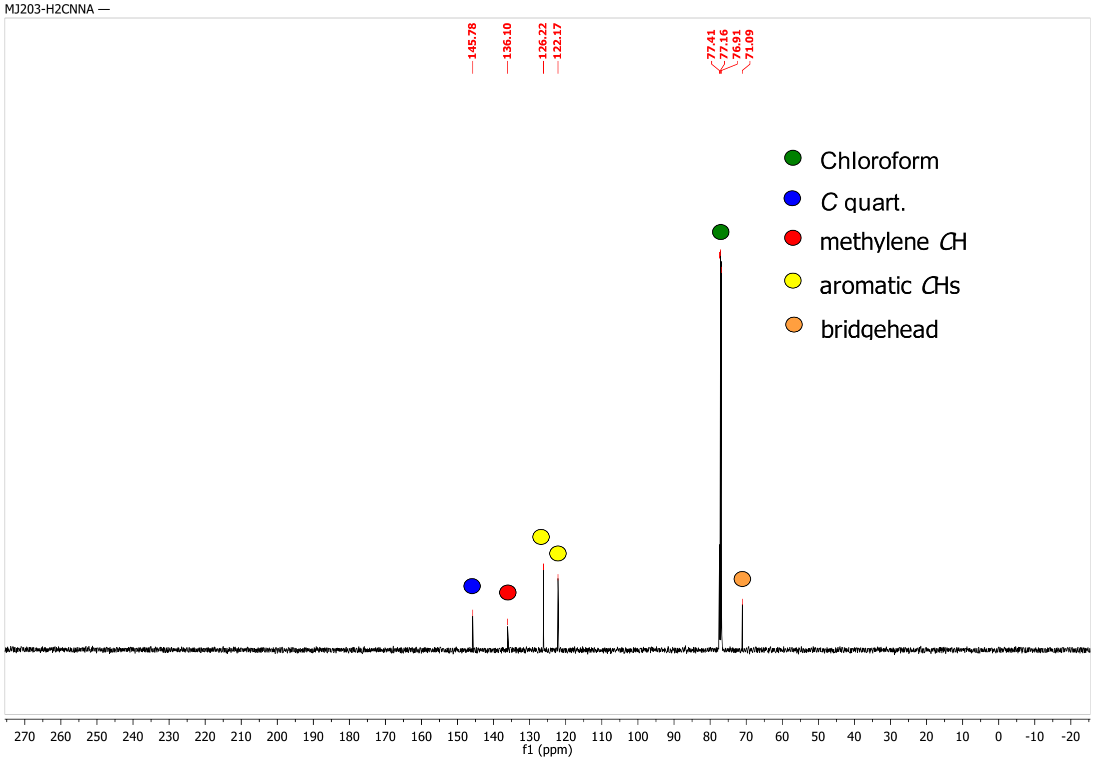

Figure S.3: ^{13}C{^{1}H} NMR spectrum (chloroform-d, 126 MHz, 20 °C) of **1**.

S.1.3 Reaction of 1 with H_2CPPh_3

A yellow solution of methylene triphenylphosphorane (10 mg, 0.036 mmol, 1.0 equiv) in benzene-d_6 (0.4 ml) was added to a colorless solution of **1** (8.8 mg, 0.040 mmol, 1.1 equiv) in benzene-d_6 (0.4 ml). An immediate color change to red, then red-brown was observed. The mixture was transferred to an NMR tube. After 12 h, the color of the mixture changed to dark-green to black. The contents of the tube were analyzed by ^{1}H NMR and ^{31}P{^{1}H} NMR spectroscopy, revealing, *inter alia*, the formation of ethylene. Other identified products in the mixture were triphenylphosphine and **A**. While all of the methylene phosphorane was consumed, *ca.* 50% of **1** was still present. Performing the reaction with $H_2{}^{13}CPPh_3$ or D_2CPPh_3 resulted in formation of $H_2{}^{13}CCH_2$ and D_2CCH_2, respectively. The intricate second-order coupling pattern of $H_2{}^{13}CCH_2$ observed in Figure S.6 matches that previously reported for this species.[8] The yield of 21% was determined by integration of the ^{1}H NMR resonance for ethylene; however, this value is only approximate as some ethylene is undoubtedly lost to the headspace of the NMR sample.

S.1.4 Reaction of 1 with *N*-Heterocyclic Carbenes

a) DAC + 1: A solution of **1** (8.5 mg, 0.023 mmol, 1.0 equiv) in benzene-d_6 (0.4 ml) was added to a solution of DAC (5 mg, 0.023 mmol, 1.0 equiv) in benzene-d_6 (0.4 ml) at 25 °C. The reaction mixture was transferred to an NMR tube. ^{1}H NMR spectroscopy indicated complete conversion of **2** after 28 h and formation of the corresponding terminal olefin, which was previously characterized.[9]

b) IPr + 1: A solution of **2** (5.0 mg, 0.023 mmol, 1.1 equiv) in benzene-d_6 (0.4 ml) was added to a solution of IPr (8.7 mg, 0.023 mmol, 1.0 equiv) in benzene-d_6 (0.4 ml) at 25 °C. The reaction mixture was transferred to an NMR tube and heated to 80 °C for 13 h. ^{1}H NMR spectroscopy indicated formation of the corresponding terminal olefin (70%), which was previously characterized.[10]

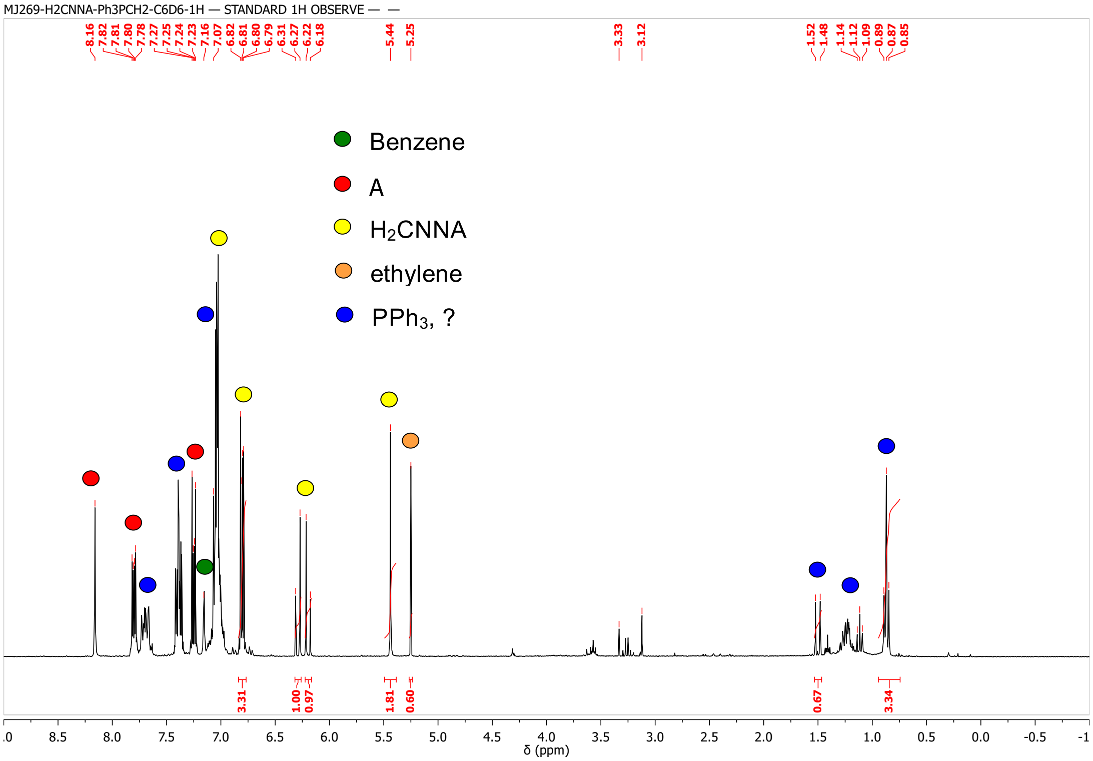

Figure S.4: ^{1}H NMR spectrum (benzene-d_6, 300 MHz, 20 °C) of the reaction mixture of **1** and H_2CPPh_3.

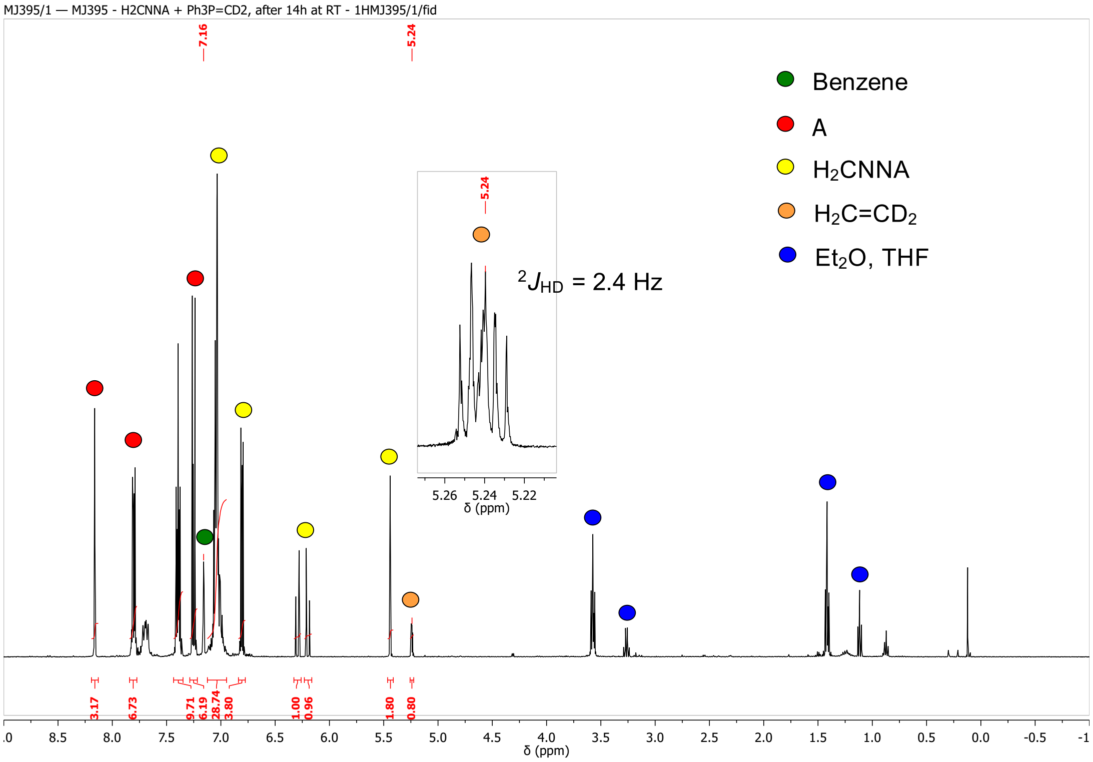

Figure S.5: ^{1}H NMR spectrum (benzene-d_6, 400 MHz, 20 °C) of the reaction mixture of **1** and D_2CPPh_3.

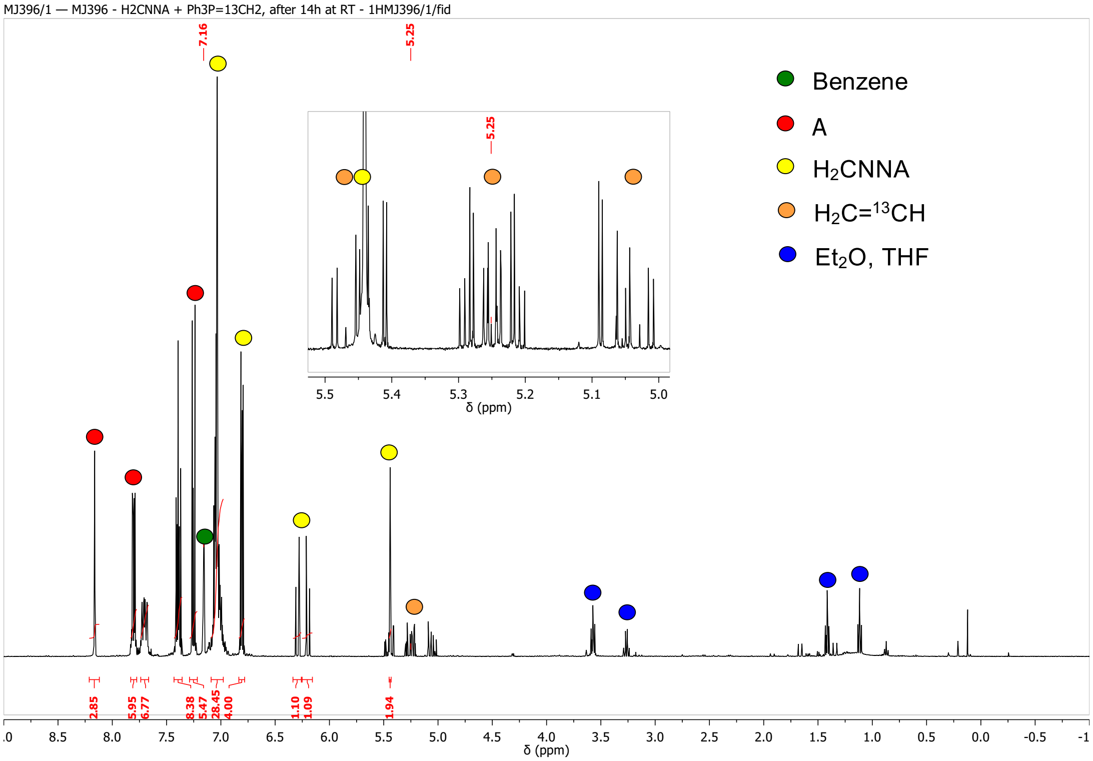

Figure S.6: ^{1}H NMR spectrum (benzene-d_6, 400 MHz, 20 °C) of the reaction mixture of **1** and $H_2{}^{13}CPPh_3$. The second-order coupling pattern matches that known in the literature.[8]

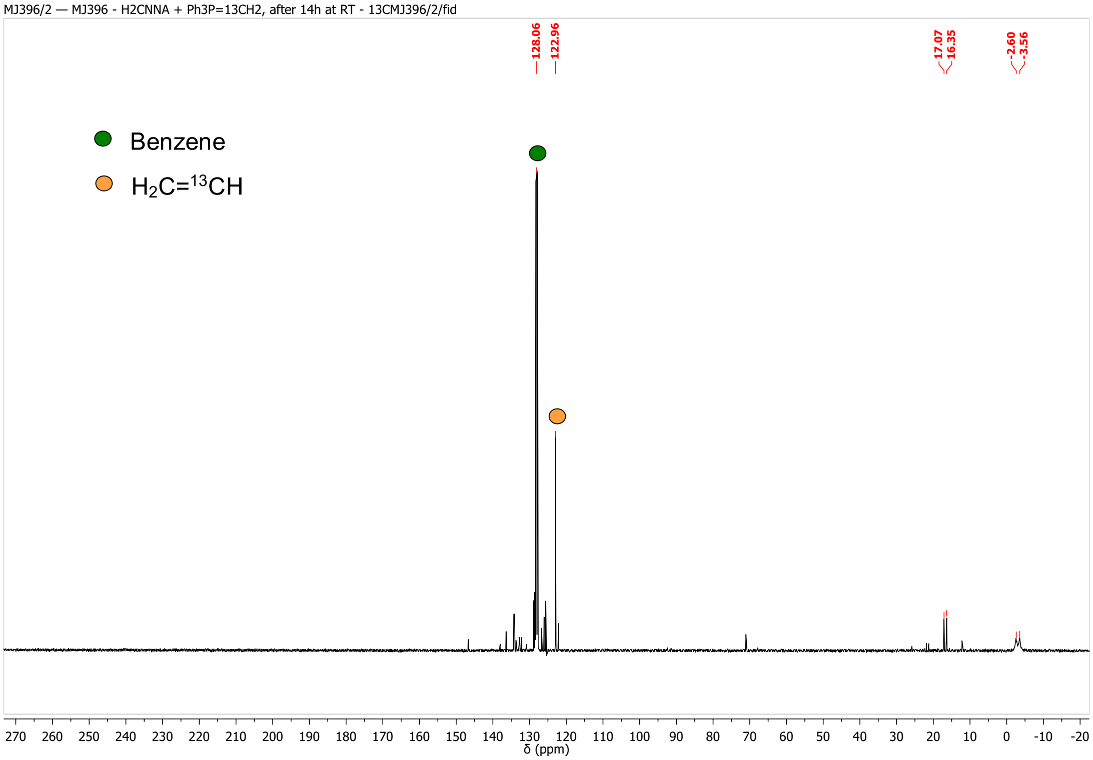

Figure S.7: ^{13}C{^{1}H} NMR spectrum (chloroform-d, 101 MHz, 20 °C) of the reaction mixture of **1** and $H_2{}^{13}CPPh_3$.

S.1.5 Synthesis of 2=CH$_2$

a) Employing 1 as the methylene source: A solution of **1** (49.2 mg, 0.22 mmol, 10.0 equiv) in benzene-d_6 (0.4 mL) was added to a solution of **2** (20 mg, 0.022 mmol, 1.0 equiv) in benzene-d_6 (0.4 mL). The dark-greenish reaction mixture was transferred to an NMR tube, which was then heated for 35 h in an oil bath at 55 °C. Upon heating, the color of the reaction mixture changed to red-brown. The tube was brought back into the glovebox, its contents transferred to a scintillation vial and volatiles were removed under vacuum. The orange residue was extracted with pentane (2 × 1 mL), the extracts filtered through a glassfiber filter and the bright orange solution was stored at –35 °C. Orange needles formed overnight which were isolated and dried under vacuum to give the title compound. Yield: 6.3 mg (0.0070 mmol, 32%).

b) Employing H$_2$CPPh$_3$ as the methylene source: A solution of methylene triphenylphosphorane (30.9 mg, 0.110 mmol, 2.0 equiv) in THF (3 mL) was slowly added at 25 °C to a solution of **2** (50.0 mg, 0.055 mmol, 1.0 equiv) in THF (3 mL). The reaction mixture was stirred for 30 min resulting in a color change to golden-yellow. A solution of lutidinium triflate (14.4 mg, 0.055 mmol, 1.0 equiv) in THF (1 mL) was added to the reaction mixture at 25 °C and the resulting reaction mixture was stirred for a further 5 min. Upon addition of the lutidinium triflate, a rapid color change to red was observed. All volatile materials were removed from the reaction mixture under reduced pressure. The orange-brown remaining solid was extracted with pentane (3 × 1 mL). All volatile materials were removed from the combined pentane extracts under vacuum. The orange solid was dissolved in pentane (2 mL), and the solution filtered through a glassfiber filter. The filtrate was stored overnight at –35 °C. Dark-orange crystals formed, which were separated from the supernatant and dried under vacuum to give the title compound. Yield: 44.0 mg (0.049 mmol, 88%). This material did not pass elemental analysis, possibly due to incomplete combustion.

Elem. Anal. Found(Calc'd) for C$_{49}$H$_{70}$O$_4$W: C 63.10 (64.89), H 7.83 (7.78); ^{1}H NMR (benzene-d_6, 500 MHz, 20 °C) δ 8.95 (s, 2H, $^2J_{\mathrm{WH}}$ = 156.0 Hz, $^1J_{\mathrm{CH}}$ = 155.6 Hz, W=CH$_2$), 7.01 (d, $^3J_{\mathrm{HH}}$ = 7.8 Hz, 8H, H$_{\text{Ar-meta}}$ ODipp), 6.87 (t, $^3J_{\mathrm{HH}}$ = 7.8 Hz, 4H, H$_{\text{Ar-para}}$ ODipp), 3.71 (sept, $^3J_{\mathrm{HH}}$ = 6.7 Hz, 8H, CH(CH$_3$)$_2$), 1.09 (d, $^3J_{\mathrm{HH}}$ = 6.7 Hz, 48H, CH(CH_3)$_2$); ^{13}C{^{1}H} NMR (benzene-d_6, 126 MHz, 20 °C) δ 232.9 (s, $^1J_{\mathrm{WC}}$ = 185.0 Hz, W=CH$_2$), 158.0 (s, C$_{\text{ipso}}$ ODipp), 138.6 (s, C$_{\text{ortho}}$ ODipp), 123.8 (s, C$_{\text{meta}}$ ODipp), 123.7 (s, C$_{\text{para}}$ ODipp), 26.9 (s, CH(CH$_3$)$_2$), 23.9 (s, CH(CH$_3$)$_2$).

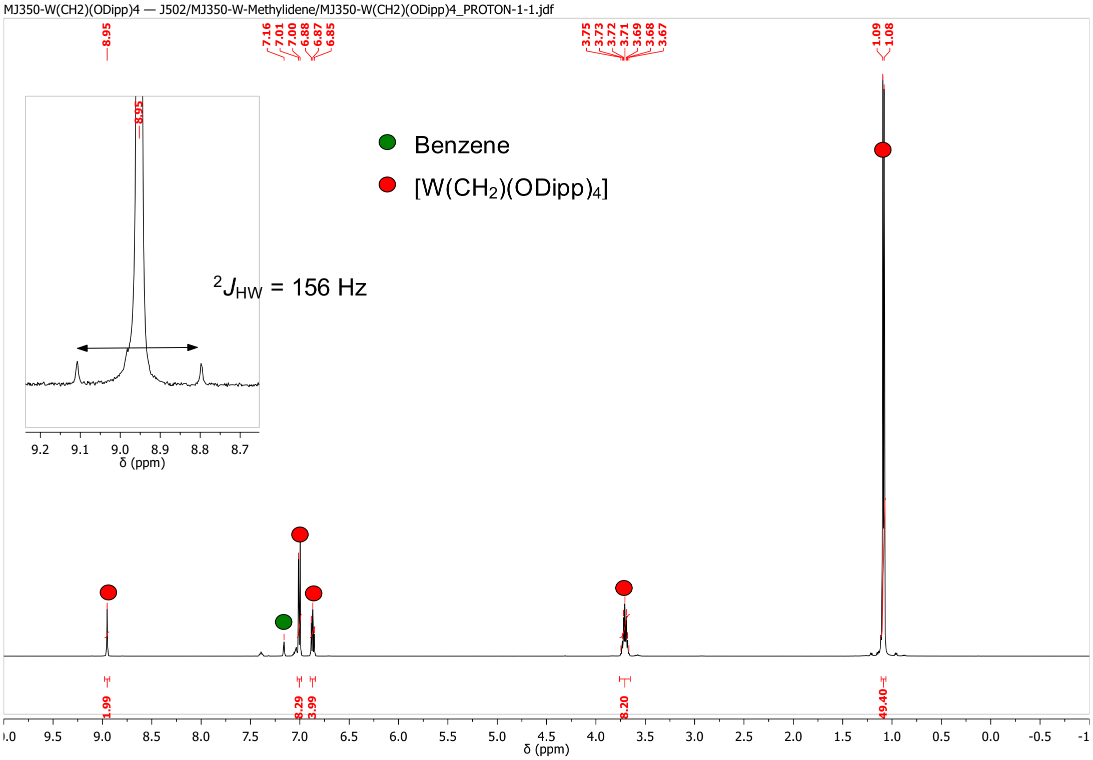

Figure S.8: ^{1}H NMR spectrum (THF-d_8, 500 MHz, 20 °C) of **2**=CH_2.

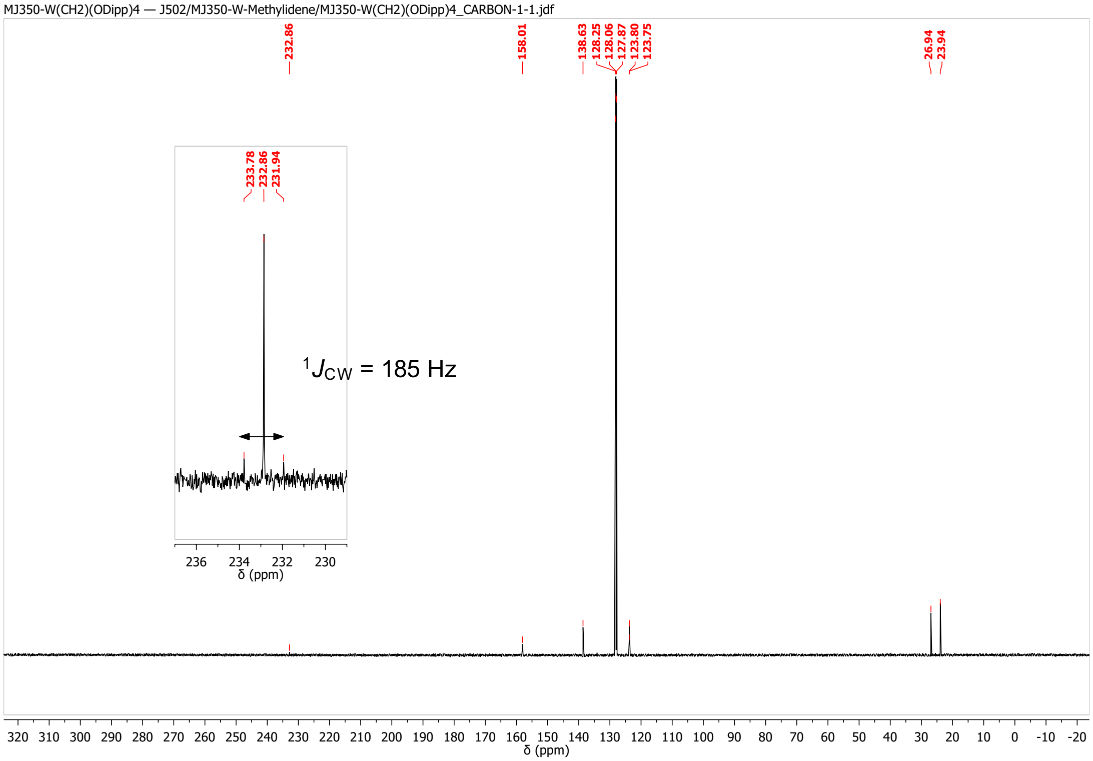

Figure S.9: ^{13}C{^{1}H} NMR spectrum (THF-d_8, 126 MHz, 20 °C) of **2**=CH_2.

S.1.6 Synthesis of [$MePPh_3$][**2**≡CH]

A solution of methylene triphenylphosphorane (61.9 mg, 0.220 mmol, 2.0 equiv) in THF (1.5 mL) was slowly added at 25 °C to a solution of **2** (100 mg, 0.110 mmol, 1.0 equiv) in THF (1.5 mL). The reaction mixture was stirred for 30 min resulting in a color change to golden-yellow. All volatile materials were removed from the reaction mixture under vacuum. The remaining orange-brown solid was washed with pentane (3 × 2 mL), redissolved in THF (*ca.* 1 mL) and pentane was slowly added until the solution turned slightly cloudy. This mixture was filtered through a glassfiber filter, and the filtrate was stored for 48 h at –35 °C resulting in formation of dark-yellow crystals which were isolated, washed with cold pentane (1 mL) and dried under vacuum giving the title compound. Yield: 63.8 mg (0.054 mmol, 49%). In order to determine the $^1J_{WC}$ coupling constant by $^{13}C\{^1H\}$ NMR spectroscopy, the ^{13}C-labelled methylidyne complex was prepared analogously using $H_2{}^{13}CPPh_3$.

Elem. Anal. Found(Calc'd) for $C_{68}H_{87}O_4PW$: C 68.45 (69.03), H 7.56 (7.41); ^1H NMR (THF-d_8, 500 MHz, 20 °C) δ 7.85–7.62 (m, 15H, H_{Ar} PPh), 6.76 (d, $^3J_{HH}$ = 7.7 Hz, 8H, $H_{Ar\text{-}meta}$ ODipp), 6.44 (t, $^3J_{HH}$ = 7.7 Hz, 4H, $H_{Ar\text{-}para}$ ODipp), 4.36 (s, 1H, $^2J_{WH}$ = 78.0 Hz, $^1J_{CH}$ = 168.0 Hz, W≡CH), 4.10 (sept, $^3J_{HH}$ = 6.8 Hz, 8H, CH(CH_3)$_2$), 2.92(d, $^2J_{PH}$ = 13.8 Hz, 3H, PCH_3), 0.86 (d, $^3J_{HH}$ = 6.8 Hz, 48H, CH(CH_3)$_2$; $^{13}C\{^1H\}$ NMR (THF-d_8, 126 MHz, 20 °C) δ 262.1 (s, $^1J_{WC}$ = 290.1 Hz, W≡CH), 161.3 (s, C_{ipso} ODipp), 138.4 (s, C_{ortho} ODipp), 135.8 (s, aryl PPh_3Me), 134.0 (d, J_{PC} = 9.8 Hz, aryl PPh_3Me), 131.0 (d, J_{PC} = 13.3 Hz, aryl PPh_3Me), 122.7 (s, C_{meta} ODipp), 120.5 (d, J_{PC} = 89.0 Hz, aryl PPh_3Me), 118.0 (s, C_{para} ODipp), 25.7 (s, CH(CH_3)$_2$), 24.4 (s, CH(CH$_3$)$_2$, 8.5 (d, $^1J_{PC}$ = 55.3 Hz, methyl PPh_3Me); $^{31}P\{^1H\}$ NMR (THF-d_8, 203 MHz, 20 °C) δ 22.7 (s, $MePPh_3$).

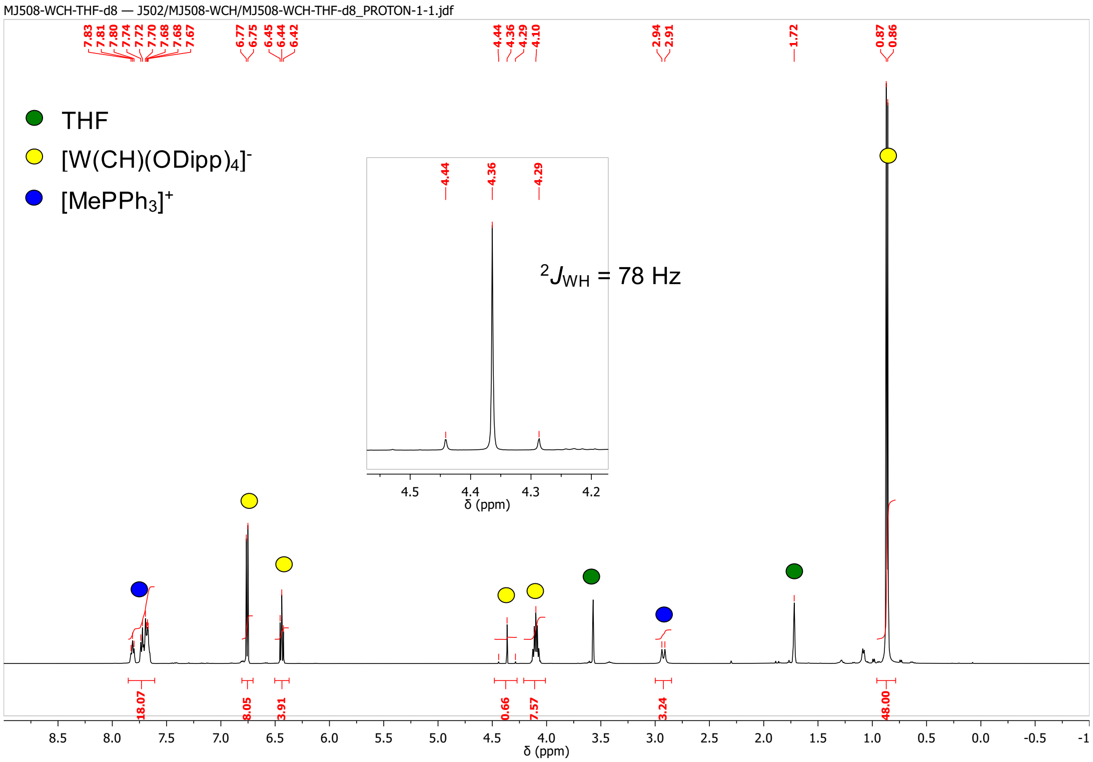

Figure S.10: ^{1}H NMR spectrum (THF-d_8, 500 MHz, 20 °C) of [MePPh$_3$][**2**≡CH].

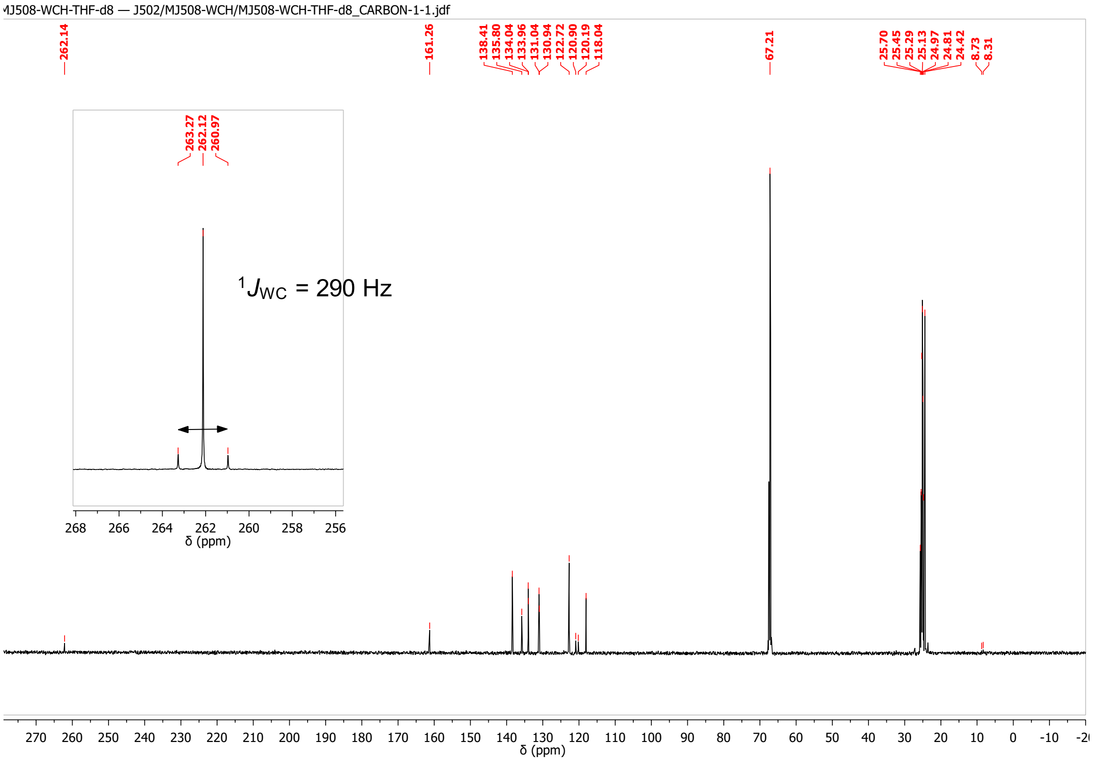

Figure S.11: $^{13}C\{^1H\}$ NMR spectrum (THF-d_8, 126 MHz, 20 °C) of [$MePPh_3$][**2**≡CH]. The inset spectrum showing the W-C coupling was employing a sample of [$H_3{}^{13}CPPh_3$][W(^{13}CH)(ODipp)$_4$]

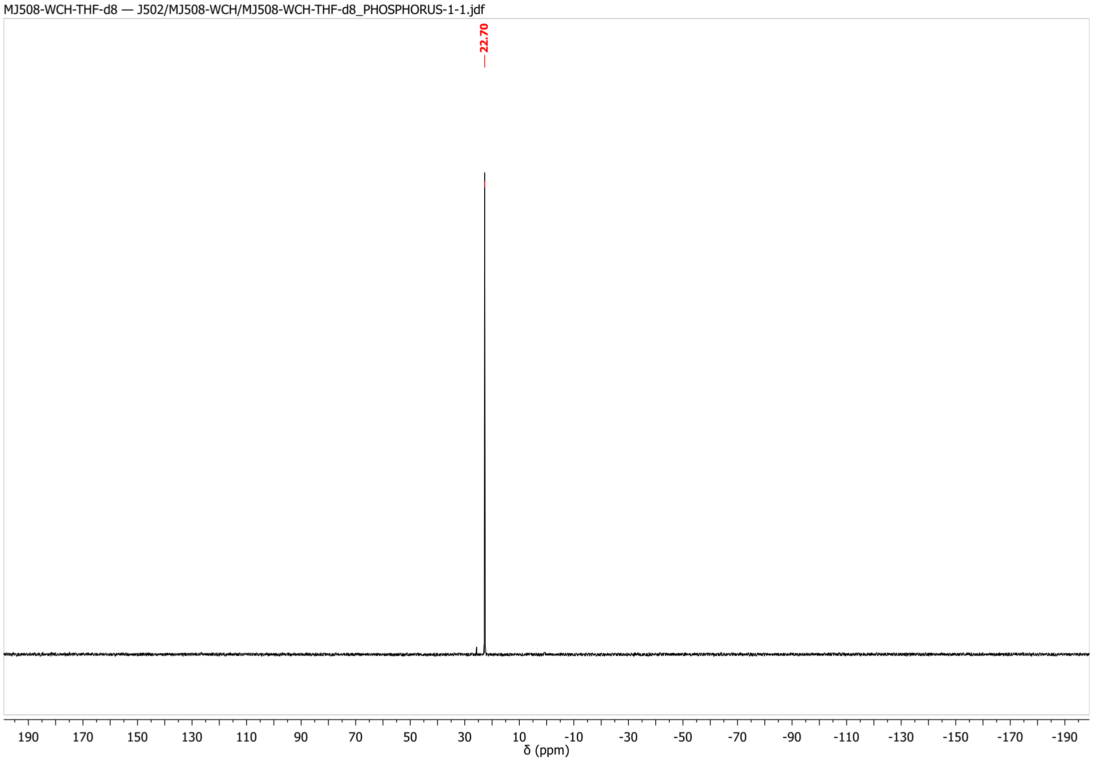

Figure S.12: $^{31}P\{^1H\}$ NMR spectrum (THF-d_8, 203 MHz, 20 °C) of [$MePPh_3$][**2**≡CH].

S.2 X-Ray Diffraction Studies

The crystals were mounted in hydrocarbon oil on a nylon loop or a glass fiber. Low-temperature (100 K) data were collected on a Bruker-AXS X8 Kappa Duo diffractometer coupled to a Smart Apex2 CCD detector with Mo Kα radiation ($\lambda = 0.71073$ Å) with ϕ- and ω-scans. A semi-empirical absorption correction was applied to the diffraction data using SADABS.[11] All structures were solved by intrinsic phasing using SHELXT[12] and refined against F^2 on all data by full-matrix least squares with SHELXL-2015[13] using established methods.[14] All non-hydrogen atoms were refined anisotropically. All hydrogen atoms were included in the model at geometrically calculated positions and refined using a riding model. The isotropic displacement parameters of all hydrogen atoms were fixed to 1.2 times the U_{eq} value of the atoms they are linked to (1.5 times for methyl groups). Descriptions of the individual refinements follow below and details of the data quality and a summary of the residual values of the refinements for all structures are given in Table S.1. Further details can be found in the form of .cif files available from the CCDC.

Crystals of **1** suitable for an X-ray diffraction analysis were grown from a concentrated solution in diethyl ether at −30 °C. **1** crystallized in the monoclinic space group $C2/c$ with one molecule in the asymmetric unit and no solvents of crystallization. The methylene hydrogen atoms were placed at geometrically calculated positions rather than those from the difference electron Fourier map.

Crystals of **2**=CH_2 suitable for an X-ray diffraction analysis were grown overnight from a concentrated solution in pentane at −35 °C. **2**=CH_2 crystallized in the orthorhombic space group $P2_12_12$ with half a molecule of **2**=CH_2 and half a molecule of pentane per asymmetric unit. Both species were modelled as whole molecules constrained to 50% occupancy to ignore the symmetry operation generating the other half. Due to this high degree of inherent disorder in the refinement model, similarity restraints and rigid bond restraints on anisoptropic displacement parameters were applied uniformly. A two-part up–down disorder of the central tungsten methylidene unit was freely refined to a ratio of 0.8540(6) with the help of similarity restraints on 1–2 distances for tungsten-oxygen and tungsten-carbon distances; see Figure S.13 for an illustration. The anisotropic displacement parameters of the methylidene carbon of the minor part was constrained to match those of the major part to remove refinement instabilities. Due to poor data quality, the methylidene hydrogen atoms could not be located in the difference electron density map, so were positioned at

geometrically calculated positions. The isopropyl groups were refined with the help of both 1–2 and 1–3 distance similarity restraints. The crystal was merohedrally twinned with a batch scale factor (BASF) freely refined to 0.240(8).

Crystals of [$MePPh_3$][**2**≡CH] suitable for an X-ray diffraction analysis were grown at 25 °C from a concentrated benzene solution at 25 °C. [$MePPh_3$][**2**≡CH] crystallized in the triclinic space group $P\overline{1}$ with one cation–anion pair per asymmetric unit. One isopropyl group in one of the four 2,6-diisopropylphenoxide ligands was modelled as disordered over two positions with the help of similarity restraints on 1–2 and 1–3 distances along with similarity restraints and rigid bond restraints on anisotropic displacement parameters. The disorder ratio refined freely to 0.820(8). The methylidyne hydrogen was located in the difference electron density Fourier map, and its position was refined semi-freely using a distance restraint.

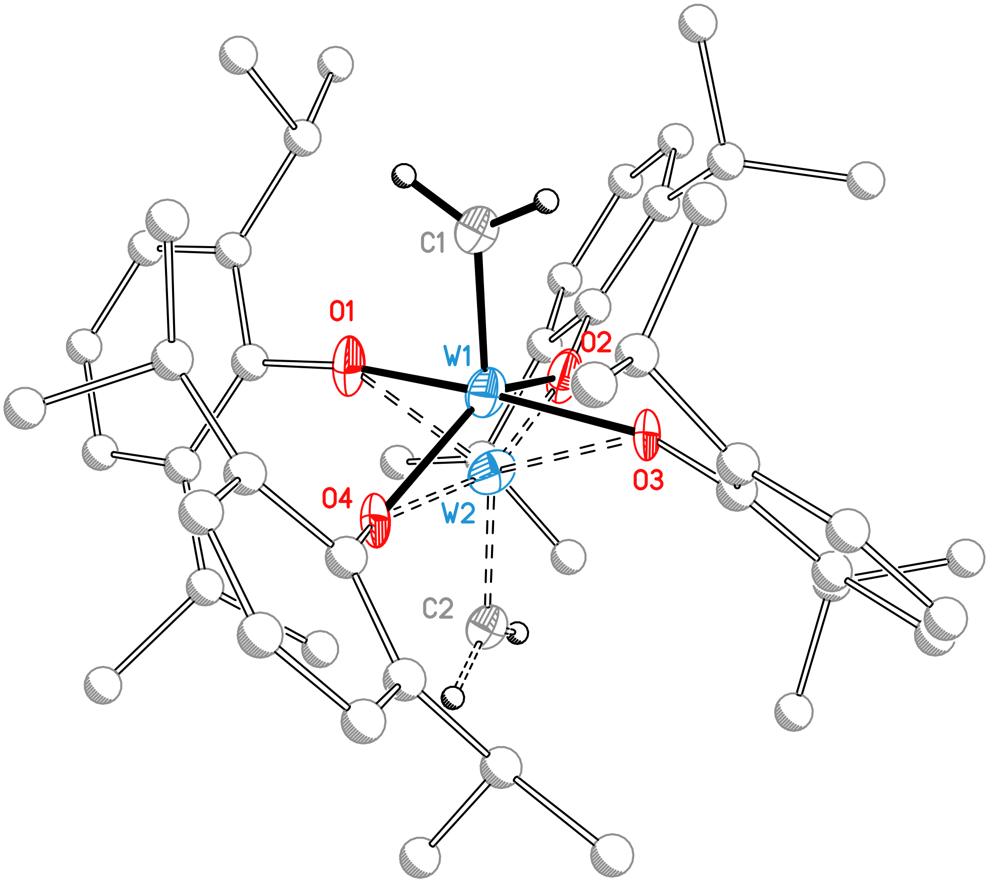

Figure S.13: An illustration of the methylidene disorder modelled in the refinement of the crystal structure of **2**=CH_2. For clarity, 50% probability thermal ellipsoids are only shown for the disordered unit. The rendering in the main manuscript shows all thermal ellipsoids.

Table S.1: Crystallographic Data for **1** and **2**=CH_2

	**1**	**2**=CH_2	[$MePPh_3$][**2**≡CH]
Reciprocal Net code / CCDC	X8_16197 / CCDC 1580349	X8_17012 / CCDC 1580350	X8_16249 / CCDC 1580351
Empirical formula, FW (g/mol)	$C_{15}H_{12}N_2$, 220.27	$C_{27}H_{40.50}O_25W_{0.5}$, 489.02	$C_{68}H_{87}O_4PW$, 1183.19
Color / Morphology	Colorless / Plate	Orange / Plate	Orange / Block
Crystal size (mm^3)	$0.200 \times 0.100 \times 0.055$	$0.312 \times 0.169 \times 0.069$	$0.218 \times 0.126 \times 0.079$
Temperature (K)	100(2)	100(2)	100(2)
Wavelength (Å)	0.71073	0.71073	0.71073
Crystal system, Space group	Monoclinic, $C2/c$	Orthorombic, $P2_12_12$	Triclinic, $P\bar{1}$
Unit cell dimensions (Å, °)	$a = 22.851(3)$, $\alpha = 90$	$a = 15.0937(12)$, $\alpha = 90$	$a = 13.4644(6)$, $\alpha = 106.468(2)$
	$b = 5.7145(6)$, $\beta = 98.174(3)$	$b = 17.6924(14)$, $\beta = 90$	$b = 13.7709(6)$, $\beta = 110.976(2)$
	$c = 16.970(2)$, $\gamma = 90$	$c = 9.6626(8)$, $\gamma = 90$	$c = 18.7517(8)$, $\gamma = 96.554(2)$
Volume (Å^3)	2193.6(5)	2580.3(4)	3021.7(2)
Z	8	4	2
Density (calc., g/cm^3)	1.334	1.259	1.300
Absorption coefficient (mm^{-1})	0.080	2.279	1.984
$F(000)$	928	1022	1232
Theta range for data collection (°)	1.801 to 30.454	1.773 to 33.191	1.241 to 37.166
Index ranges	$-32 \le h \le 32$, $-8 \le k \le 4$,	$-23 \le h \le 23$, $-27 \le k \le 27$,	$-22 \le h \le 22$, $-23 \le k \le 23$,
	$-24 \le l \le 24$	$-13 \le l \le 14$	$-31 \le l \le 31$
Reflections collected	20936	114430	344550
Independent reflections, R_{int}	3320, 0.0416	9879, 0.0463	30460, 0.0449
Completeness to $\theta_{\max}$ (%)	99.8	100.0	100.0
Absorption correction	Semi-empirical from equiv.	Semi-empirical from equiv.	Semi-empirical from equiv.
Refinement method	Full-matrix least-squares on F^2	Full-matrix least-squares on F^2	Full-matrix least-squares on F^2
Data / Restraints / Parameters	3320 / 0 / 154	9879 / 1256 / 531	30460 / 44 / 709
Goodness-of-fit[a]	1.048	1.098	1.049
Final R indices[b] $[I > 2\sigma(I)]$	$R_1 = 0.0474$, $wR_2 = 0.1199$	$R_1 = 0.0265$, $wR_2 = 0.0575$	$R_1 = 0.0257$, $wR_2 = 0.0588$
R indices[b] (all data)	$R_1 = 0.0590$, $wR_2 = 0.1298$	$R_1 = 0.0346$, $wR_2 = 0.0604$	$R_1 = 0.0337$, $wR_2 = 0.0616$
Largest diff. peak and hole (e·Å^{-3})	0.410 and −0.302	1.258 and −1.643	1.784 and −1.812

[a] GooF = $\sqrt{\frac{\Sigma[w(F_o^2-F_c^2)^2]}{(n-p)}}$ [b] $R_1 = \frac{\Sigma||F_o|-|F_c||}{\Sigma|F_o|}$; $wR_2 = \sqrt{\frac{\Sigma[w(F_o^2-F_c^2)^2]}{\Sigma[w(F_o^2)^2]}}$; $w = \frac{1}{\sigma^2(F_o^2)+(aP)^2+bP}$; $P = \frac{2F_c^2+\max(F_o^2,0)}{3}$

References

[1] A. B. Pangborn, M. A. Giardello, R. H. Grubbs, R. K. Rosen and F. J. Timmers, *Organometallics*, 1996, **15**, 1518–1520.

[2] D. B. G. Williams and M. Lawton, *J. Org. Chem.*, 2010, **75**, 8351–8354.

[3] L. A. Carpino, R. E. Padykula, D. E. Barr, F. H. Hall, J. G. Krause, R. F. Dufresne and C. J. Thoman, *J. Org. Chem.*, 1988, **53**, 2565–2572.

[4] T. W. Hudnall, J. P. Moerdyk and C. W. Bielawski, *Chem. Commun.*, 2010, **46**, 4288.

[5] (*a*) M. L. Listemann, J. C. Dewan and R. R. Schrock, *J. Am. Chem. Soc.*, 1985, **107**, 7207–7208; (*b*) M. L. Listemann, R. R. Schrock, J. C. Dewan and R. M. Kolodziej, *Inorg. Chem.*, 1988, **27**, 264–271.

[6] H. J. Bestmann, W. Stransky and O. Vostrowsky, *Chem. Ber.*, 1976, **109**, 1694–1700.

[7] G. R. Fulmer, A. J. M. Miller, N. H. Sherden, H. E. Gottlieb, A. Nudelman, B. M. Stoltz, J. E. Bercaw and K. I. Goldberg, *Organometallics*, 2010, **29**, 2176–2179.

[8] R. M. Lynden-Bell and N. Sheppard, *Proc. Royal Soc. A*, 1962, **269**, 385–403.

[9] D. T. Chase, J. P. Moerdyk and C. W. Bielawski, *Org. Lett.*, 2014, **16**, 812–815.

[10] K. Powers, C. Hering-Junghans, R. McDonald, M. J. Ferguson and E. Rivard, *Polyhedron*, 2016, **108**, 8–14.

[11] (*a*) *Bruker, SADABS, 2008*; (*b*) L. Krause, R. Herbst-Irmer, G. M. Sheldrick and D. Stalke, *J. Appl. Cryst.*, 2015, **48**, 3–10.

[12] (*a*) G. M. Sheldrick, *Acta Crystallogr. A*, 2008, **64**, 112–122; (*b*) G. M. Sheldrick, *Acta Crystallogr. A*, 2015, **71**, 3–8.

[13] G. M. Sheldrick and T. R. Schneider, *Methods in Enzymology*, 1997, **277**, 319–343.

[14] P. Müller, *Crystallogr. Rev.*, 2009, **15**, 57–83.
